# The genome sequence of the Lichen Button, *Acleris literana* (Linnaeus, 1758)

**DOI:** 10.12688/wellcomeopenres.19481.1

**Published:** 2023-06-02

**Authors:** Liam M. Crowley, James Hammond, University of Oxford and Wytham Woods Genome Acquisition Lab, Darwin Tree of Life Barcoding collective, Wellcome Sanger Institute Tree of Life programme, Wellcome Sanger Institute Scientific Operations: DNA Pipelines collective, Tree of Life Core Informatics collective, Darwin Tree of Life Consortium

**Affiliations:** 1 University of Oxford, Oxford, England, UK

**Keywords:** Acleris literana, Lichen Button, genome sequence, chromosomal, Lepidoptera

## Abstract

We present a genome assembly from an individual male *Acleris literana* (the Lichen Button; Arthropoda; Insecta; Lepidoptera; Tortricidae). The genome sequence is 674.9 megabases in span. Most of the assembly is scaffolded into 30 chromosomal pseudomolecules, including the Z sex chromosome. The mitochondrial genome has also been assembled and is 16.4 kilobases in length. Gene annotation of this assembly on Ensembl identified 12,577 protein coding genes.

## Species taxonomy

Eukaryota; Metazoa; Ecdysozoa; Arthropoda; Hexapoda; Insecta; Pterygota; Neoptera; Endopterygota; Lepidoptera; Glossata; Ditrysia; Tortricoidea; Tortricidae; Tortricinae; *Acleris*; *Acleris literana* (Linnaeus, 1758) (NCBI:txid1100899).

## Background

*Acleris literana* (Linnaeus, 1758), also known as the Lichen Button or Sprinkled Rough-wing, is a moth in the Tortricidae family. The species’ vernacular names reference the cryptic colouration of the forewings, which often exhibits attractive black and blue-green patterning, resembling the surface of a lichen and raised scales giving the wing a ‘rough’ appearance. Like other members of its genus which overwinter in the adult stage, the species is extremely polymorphic, and forewing colouration can vary in the degree of blue colouring, the quantity and distribution of raised scales, and appearance of brown colouring: in some cases, brown colouration totally replaced the green ground-colour of the forewing ( Bradley, 1962). Lichen-like colouring of the forewing is shared with species in other lepidopteran families, such as the noctuids *Nyctobrya muralis* and *Griposia aprilina*, or the larva of the geometrid *Odontopera bidentata*, and is an example of convergent evolution.

The species’ high degree of polymorphism may be partially explained by apostatic selection, as copulation and egg-laying occur in spring ( Bradley *et al.*, 1973; Elliott *et al.*, 2018), and thus there is strong selective pressure on adults to survive the winter. The dominant foodplant for this species in the British Isles is oak ( *Quercus*), however in captivity the larvae accept a range of other deciduous trees ( Bradley *et al.*, 1973; Elliott *et al.*, 2018). Larvae feed between spun leaves from May to June, and pupate between June and July. Adults emerge from August and are on the wing into October, after which they hibernate until they re-emerge in the following spring. The species is found locally across the British Isles, being most abundant in oak woodland ( Bradley *et al.*, 1973). Globally, the species is confined to Europe and Asia Minor ( Hancock *et al.*, 2015).

A genome sequence for *Acleris literana* will contribute to our understanding of the genomic basis of polymorphism and the evolution of cryptic coloration in Lepidoptera. The genome of *Acleris literana* was sequenced as part of the Darwin Tree of Life Project, a collaborative effort to sequence all named eukaryotic species in the Atlantic Archipelago of Britain and Ireland. Here we present a chromosomally complete genome sequence for *Acleris literana* based on one male specimen of the form *A. literana* ab. *squamana* (Fabricius, 1775), from Wytham Woods, Oxfordshire, UK.

## Genome sequence report

**Figure 1.**
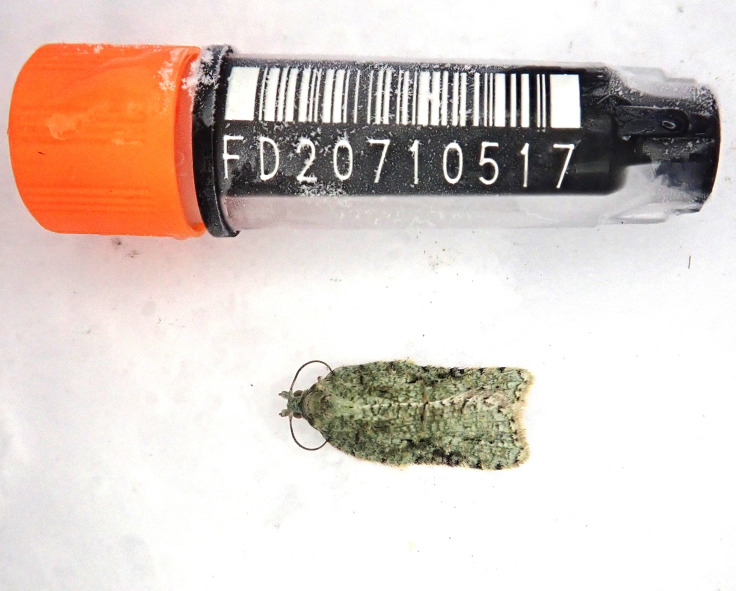
Photograph of the *Acleris literana* (ilAclLite1) specimen used for genome sequencing.

The genome was sequenced from one male *Acleris literana* ( Figure 1) collected from Wytham Woods, Oxfordshire, UK (latitude 51.77, longitude –2.34). A total of 29-fold coverage in Pacific Biosciences single-molecule HiFi long reads was generated. Primary assembly contigs were scaffolded with chromosome conformation Hi-C data. Manual assembly curation corrected 15 missing joins or mis-joins and removed six haplotypic duplications, reducing the assembly length by 0.38% and the scaffold number by 4.88%.

**Figure 2.**
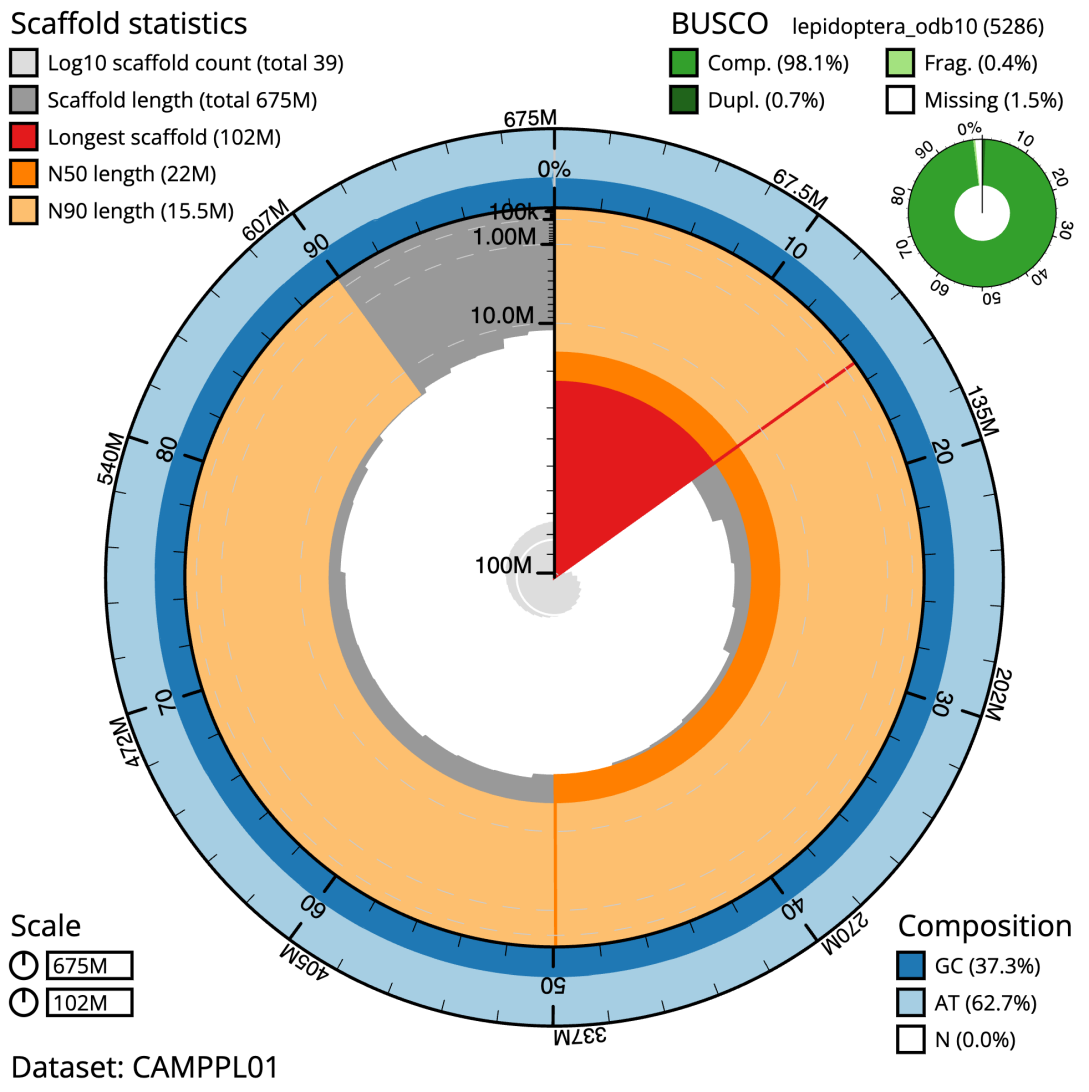
Genome assembly of *Acleris literana*, ilAclLite1.1: metrics. The BlobToolKit Snailplot shows N50 metrics and BUSCO gene completeness. The main plot is divided into 1,000 size-ordered bins around the circumference with each bin representing 0.1% of the 674,889,359 bp assembly. The distribution of scaffold lengths is shown in dark grey with the plot radius scaled to the longest scaffold present in the assembly (102,365,968 bp, shown in red). Orange and pale-orange arcs show the N50 and N90 scaffold lengths (21,986,359 and 15,473,039 bp), respectively. The pale grey spiral shows the cumulative scaffold count on a log scale with white scale lines showing successive orders of magnitude. The blue and pale-blue area around the outside of the plot shows the distribution of GC, AT and N percentages in the same bins as the inner plot. A summary of complete, fragmented, duplicated and missing BUSCO genes in the lepidoptera_odb10 set is shown in the top right. An interactive version of this figure is available at https://blobtoolkit.genomehubs.org/view/ilAclLite1.1/dataset/CAMPPL01/snail.

The final assembly has a total length of 674.9 Mb in 39 sequence scaffolds with a scaffold N50 of 22.0 Mb ( Table 1). Most (99.97%) of the assembly sequence was assigned to 30 chromosomal-level scaffolds, representing 29 autosomes and the Z sex chromosome. Chromosome-scale scaffolds confirmed by the Hi-C data are named in order of size ( Figure 2– Figure 5; Table 2). While not fully phased, the assembly deposited is of one haplotype. Contigs corresponding to the second haplotype have also been deposited. The mitochondrial genome was also assembled and can be found as a contig within the multifasta file of the genome submission.

**Table 1.** Genome data for *Acleris literana*, ilAclLite1.1.

Project accession data		
Assembly identifier	ilAclLite1.1	
Species	*Acleris literana*	
Specimen	ilAclLite1	
NCBI taxonomy ID	1100899	
BioProject	PRJEB55019	
BioSample ID	SAMEA10978974	
Isolate information	ilAclLite1, male: whole organism (DNA sequencing and Hi-C scaffolding)	
Assembly metrics *		*Benchmark*
Consensus quality (QV)	66.6	*≥ 50*
*k*-mer completeness	100%	*≥ 95%*
BUSCO **	C:98.1%[S:97.4%,D:0.7%], F:0.4%,M:1.5%,n:5,286	*C ≥ 95%*
Percentage of assembly mapped to chromosomes	99.97%	*≥ 95%*
Sex chromosomes	Z chromosome	*localised homologous pairs*
Organelles	Mitochondrial genome assembled	*complete single alleles*
Raw data accessions		
PacificBiosciences SEQUEL II	ERR10008902	
Hi-C Illumina	ERR10015059	
Genome assembly		
Assembly accession	GCA_946894065.1	
*Accession of alternate haplotype*	GCA_946894075.1	
Span (Mb)	674.9	
Number of contigs	56	
Contig N50 length (Mb)	20.4	
Number of scaffolds	39	
Scaffold N50 length (Mb)	22.0	
Longest scaffold (Mb)	102.4	
Genome annotation		
Number of protein-coding genes	12,577	
Number of non-coding genes	1,640	
Number of gene transcripts	22,165	

* Assembly metric benchmarks are adapted from column VGP-2020 of “Table 1: Proposed standards and metrics for defining genome assembly quality” from ( Rhie *et al.*, 2021). ** BUSCO scores based on the lepidoptera_odb10 BUSCO set using v5.3.2. C = complete [S = single copy, D = duplicated], F = fragmented, M = missing, n = number of orthologues in comparison. A full set of BUSCO scores is available at https://blobtoolkit.genomehubs.org/view/ilAclLite1.1/dataset/CAMPPL01/busco.

**Figure 3.**
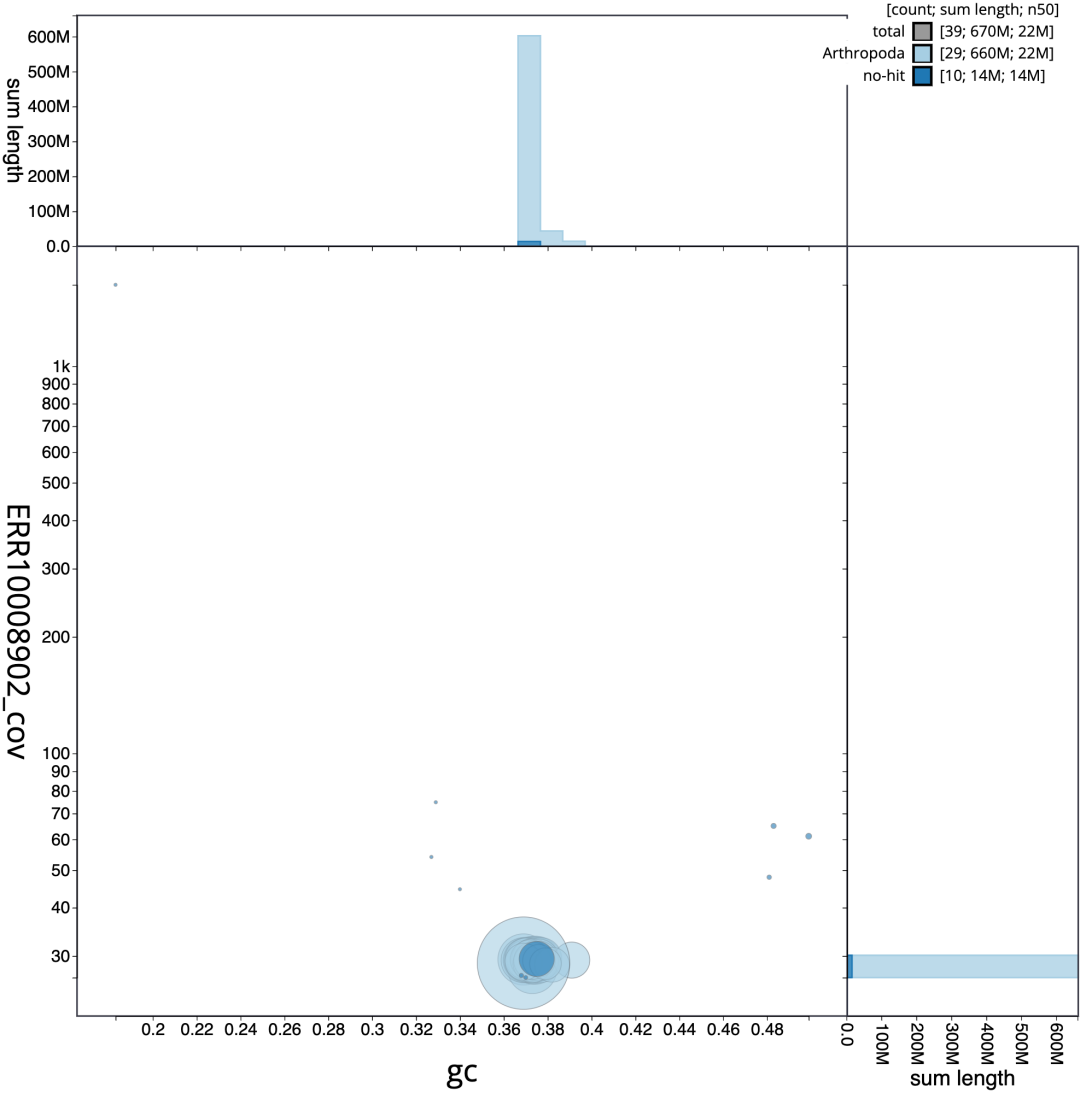
Genome assembly of *Acleris literana*, ilAclLite1.1: BlobToolKit GC-coverage plot. Scaffolds are coloured by phylum. Circles are sized in proportion to scaffold length. Histograms show the distribution of scaffold length sum along each axis. An interactive version of this figure is available at https://blobtoolkit.genomehubs.org/view/ilAclLite1.1/dataset/CAMPPL01/blob.

**Figure 4.**
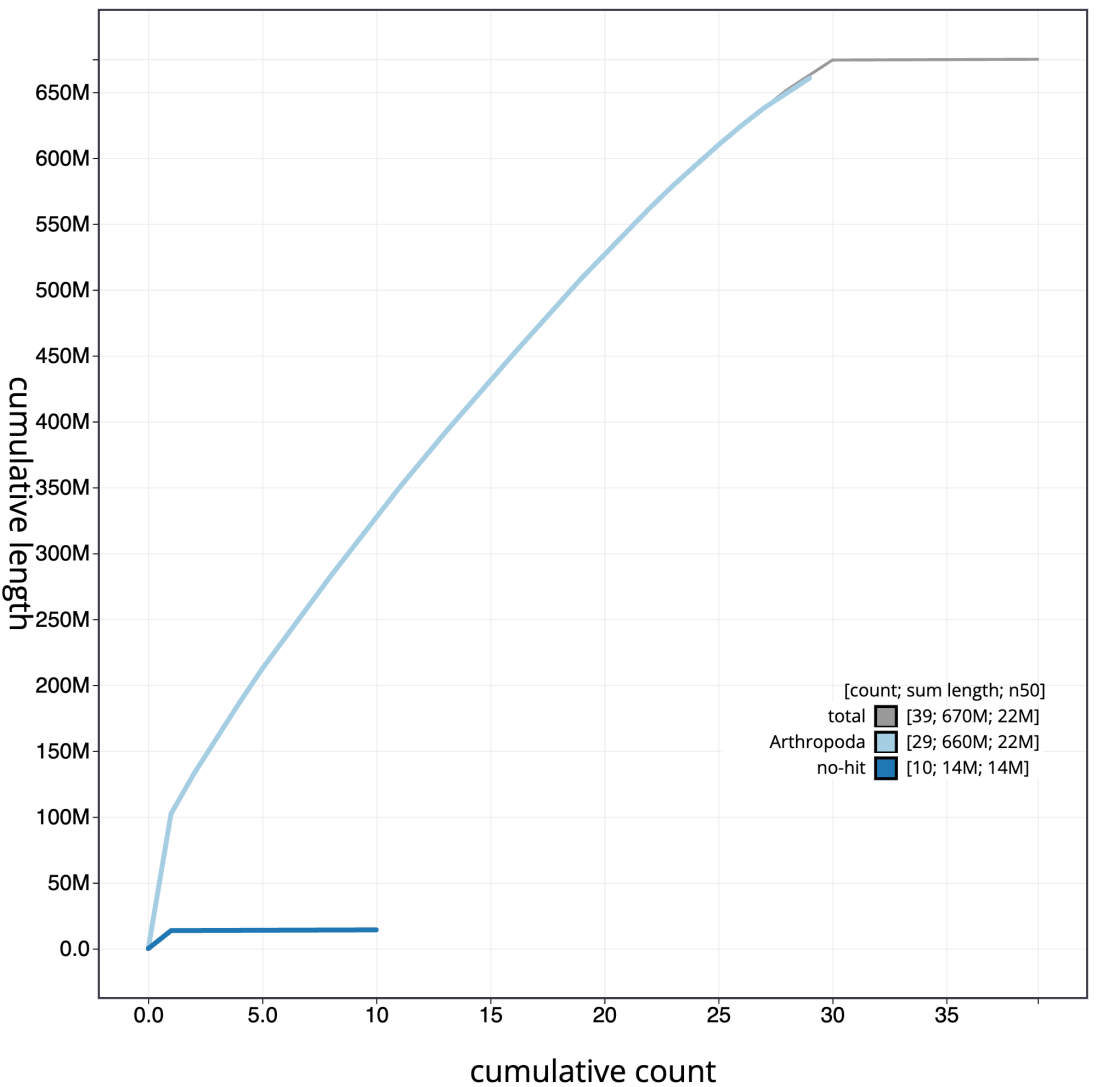
Genome assembly of *Acleris literana*, ilAclLite1.1: BlobToolKit cumulative sequence plot. The grey line shows cumulative length for all scaffolds. Coloured lines show cumulative lengths of scaffolds assigned to each phylum using the buscogenes taxrule. An interactive version of this figure is available at https://blobtoolkit.genomehubs.org/view/ilAclLite1.1/dataset/CAMPPL01/cumulative.

**Figure 5.**
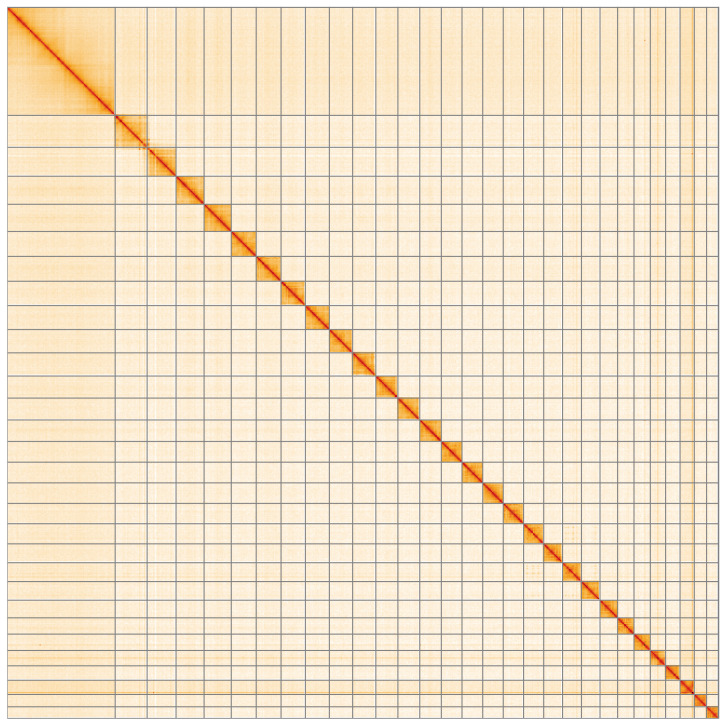
Genome assembly of *Acleris literana*, ilAclLite1.1: Hi-C contact map of the ilAclLite1.1 assembly, visualised using HiGlass. Chromosomes are shown in order of size from left to right and top to bottom. An interactive version of this figure may be viewed at https://genome-note-higlass.tol.sanger.ac.uk/l/?d=ALGJ1wFpRXSSkRYnS_iRIg.

**Table 2.** Chromosomal pseudomolecules in the genome assembly of *Acleris literana*, ilAclLite1.

INSDC accession	Chromosome	Size (Mb)	GC%
OX335669.1	1	30.28	36.9
OX335670.1	2	27.45	37.3
OX335671.1	3	26.62	37.4
OX335672.1	4	25.76	37.3
OX335673.1	5	23.65	37
OX335674.1	6	23.59	37.6
OX335675.1	7	23.04	36.9
OX335676.1	8	22.75	37.3
OX335677.1	9	22.11	37
OX335678.1	10	21.99	37.5
OX335679.1	11	20.9	37.3
OX335680.1	12	20.74	37.1
OX335681.1	13	20.43	37.4
OX335682.1	14	19.64	37
OX335683.1	15	19.64	37
OX335684.1	16	19.48	37.5
OX335685.1	17	19.27	37.7
OX335686.1	18	19.09	37.3
OX335687.1	19	17.9	37.6
OX335688.1	20	17.88	37.6
OX335689.1	21	17.63	37.2
OX335690.1	22	16.7	37.3
OX335691.1	23	15.51	37.3
OX335692.1	24	15.47	36.9
OX335693.1	25	14.53	39.1
OX335694.1	26	13.69	37.5
OX335695.1	27	13.51	38.2
OX335696.1	28	11.67	37.6
OX335697.1	29	11.04	37.9
OX335668.1	Z	102.37	36.9
OX335698.1	MT	0.02	18.5
-	unplaced	0.54	45.1

The estimated Quality Value (QV) of the final assembly is 66.6 with *k*-mer completeness of 100%, and the assembly has a BUSCO v5.3.2 completeness of 98.1% (single = 97.4%, duplicated = 0.7%), using the lepidoptera_odb10 reference set ( *n* = 5,286).

Metadata for specimens, spectral estimates, sequencing runs, contaminants and pre-curation assembly statistics can be found at https://links.tol.sanger.ac.uk/species/1100899.

## Genome annotation report

The ilAclLite1.1, GCA_946894065.1 genome assembly was annotated using the Ensembl rapid annotation pipeline ( Table 1; https://rapid.ensembl.org/Acleris_literana_GCA_946894065.1/Info/Index). The resulting annotation includes 22,165 transcribed mRNAs from 12,577 protein-coding and 1,640 non-coding genes.

## Methods

### Sample acquisition and nucleic acid extraction

A male *Acleris literana* (ilAclLite1) was reared from a pupa within a leaf spinning collected from Wytham Woods, Oxfordshire (biological vice-county Berkshire), UK (latitude 51.77, longitude –2.34) on 22 July 2021 by Liam Crowley (University of Oxford). The adult eclosed on 27 July 2021, when it was identified and snap-frozen on dry ice.

The sample was prepared for DNA extraction in the Tree of Life laboratory, Wellcome Sanger Institute (WSI). The ilAclLite1 sample was weighed and dissected on dry ice with tissue set aside for Hi-C sequencing. Whole organism tissue was disrupted using a Nippi Powermasher fitted with a BioMasher pestle. DNA was extracted at the WSI Scientific Operations core using the Qiagen MagAttract HMW DNA kit, according to the manufacturer’s instructions.

### Sequencing

Pacific Biosciences HiFi circular consensus DNA sequencing libraries were constructed according to the manufacturers’ instructions. DNA sequencing was performed by the Scientific Operations core at the WSI on the Pacific Biosciences SEQUEL II (HiFi) instrument. Hi-C data were also generated from tissue of ilAclLite1 that had been set aside, using the Arima2 kit and sequenced on the Illumina NovaSeq 6000 instrument.

### Genome assembly, curation and evaluation

Assembly was carried out with Hifiasm ( Cheng *et al.*, 2021) and haplotypic duplication was identified and removed with purge_dups ( Guan *et al.*, 2020). The assembly was then scaffolded with Hi-C data ( Rao *et al.*, 2014) using YaHS ( Zhou *et al.*, 2023). The assembly was checked for contamination as described previously ( Howe *et al.*, 2021). Manual curation was performed using HiGlass ( Kerpedjiev *et al.*, 2018) and Pretext ( Harry, 2022). The mitochondrial genome was assembled using MitoHiFi ( Uliano-Silva *et al.*, 2022), which runs MitoFinder ( Allio *et al.*, 2020) or MITOS ( Bernt *et al.*, 2013) and uses these annotations to select the final mitochondrial contig and to ensure the general quality of the sequence.

A Hi-C map for the final assembly was produced using bwa-mem2 ( Vasimuddin *et al.*, 2019) in the Cooler file format ( Abdennur & Mirny, 2020). To assess the assembly metrics, the *k*-mer completeness and QV consensus quality values were calculated in Merqury ( Rhie *et al.*, 2020). This work was done using Nextflow ( Di Tommaso *et al.*, 2017) DSL2 pipelines “sanger-tol/readmapping” ( Surana *et al.,* 2023a) and “sanger-tol/genomenote” ( Surana *et al.,* 2023b). The genome was analysed within the BlobToolKit environment ( Challis *et al.*, 2020) and BUSCO scores ( Manni *et al.*, 2021; Simão *et al.*, 2015) were calculated.

Table 3 contains a list of relevant software tool versions and sources.

**Table 3.** Software tools: versions and sources.

Software tool	Version	Source
BlobToolKit	4.0.7	https://github.com/blobtoolkit/blobtoolkit
BUSCO	5.3.2	https://gitlab.com/ezlab/busco
Hifiasm	0.16.1-r375	https://github.com/chhylp123/hifiasm
HiGlass	1.11.6	https://github.com/higlass/higlass
Merqury	MerquryFK	https://github.com/thegenemyers/MERQURY.FK
MitoHiFi	2	https://github.com/marcelauliano/MitoHiFi
PretextView	0.2	https://github.com/wtsi-hpag/PretextView
purge_dups	1.2.3	https://github.com/dfguan/purge_dups
sanger-tol/genomenote	v1.0	https://github.com/sanger-tol/genomenote
sanger-tol/readmapping	1.1.0	https://github.com/sanger-tol/readmapping/tree/1.1.0
YaHS	yahs-1.1.91eebc2	https://github.com/c-zhou/yahs

### Genome annotation

The Ensembl gene annotation system ( Aken *et al.*, 2016) was used to generate annotation for the *Acleris literana* assembly (GCA_946894065.1). Annotation was created primarily through alignment of transcriptomic data to the genome, with gap filling via protein-to-genome alignments of a select set of proteins from UniProt ( UniProt Consortium, 2019).

### Legal and ethical review process for Darwin Tree of Life Partner submitted materials

The materials that have contributed to this genome note have been supplied by a Darwin Tree of Life Partner.

The submission of materials by a Darwin Tree of Life Partner is subject to the **‘Darwin Tree of Life Project Sampling Code of Practice’**, which can be found in full on the Darwin Tree of Life website here. By agreeing with and signing up to the Sampling Code of Practice, the Darwin Tree of Life Partner agrees they will meet the legal and ethical requirements and standards set out within this document in respect of all samples acquired for, and supplied to, the Darwin Tree of Life Project.

Further, the Wellcome Sanger Institute employs a process whereby due diligence is carried out proportionate to the nature of the materials themselves, and the circumstances under which they have been/are to be collected and provided for use. The purpose of this is to address and mitigate any potential legal and/or ethical implications of receipt and use of the materials as part of the research project, and to ensure that in doing so we align with best practice wherever possible.

The overarching areas of consideration are:

- Ethical review of provenance and sourcing of the material
- Legality of collection, transfer and use (national and international)

Each transfer of samples is further undertaken according to a Research Collaboration Agreement or Material Transfer Agreement entered into by the Darwin Tree of Life Partner, Genome Research Limited (operating as the Wellcome Sanger Institute), and in some circumstances other Darwin Tree of Life collaborators.

## Data Availability

European Nucleotide Archive: *Acleris literana* (lichen button). Accession number PRJEB55019; https://identifiers.org/ena.embl/PRJEB55019. ( Wellcome Sanger Institute, 2022) The genome sequence is released openly for reuse. The *Acleris literana* genome sequencing initiative is part of the Darwin Tree of Life (DToL) project. All raw sequence data and the assembly have been deposited in INSDC databases. Raw data and assembly accession identifiers are reported in Table 1.

## Author information

Members of the University of Oxford and Wytham Woods Genome Acquisition Lab are listed here: https://doi.org/10.5281/zenodo.4789928.

Members of the Darwin Tree of Life Barcoding collective are listed here: https://doi.org/10.5281/zenodo.4893703.

Members of the Wellcome Sanger Institute Tree of Life programme are listed here: https://doi.org/10.5281/zenodo.4783585.

Members of Wellcome Sanger Institute Scientific Operations: DNA Pipelines collective are listed here: https://doi.org/10.5281/zenodo.4790455.

Members of the Tree of Life Core Informatics collective are listed here: https://doi.org/10.5281/zenodo.5013541.

Members of the Darwin Tree of Life Consortium are listed here: https://doi.org/10.5281/zenodo.4783558.
