# Supplementary material for: The genome sequence of the Lichen Button, Acleris literana (Linnaeus, 1758)
Source: Wellcome Open Res. 2023 Jun 2;8:232. [Version 1] doi: 10.12688/wellcomeopenres.19481.1 (PMC10799227; doi:10.12688/wellcomeopenres.19481.1)
Supplement: Peer review history

# Reviewer report: Reviewer response for version 1

**DOI:** 10.21956/wellcomeopenres.21579.r71111

**Published:** 2024-01-19

**Authors:** Yuttapong Thawornwattana

**Affiliations:** 1 Department of Organismic and Evolutionary Biology, Harvard University, Cambridge, MA, USA

Crowley & Hammond present a chromosome-level genome assembly of *Acleris literana* (Linnaeus, 1758) (Lepidoptera: Tortricidae), a moth collected from Wytham Woods, England.  It was generated primarily from PacBio HiFi and Hi-C data.  

 The assembly contains 29 autosomes, the Z chromosome and the mitochondrial genome.  It is of high-quality, judged by various metrics, and will be useful to the research community.  I have following comments.

 1) The expected haploid chromosome number from previous work (e.g. on karyotypes, if any) could be mentioned.  The Z chromosome appears to be several times larger than the autosomes.  Would the authors be able to comment on this, perhaps in comparison with other tortricid genomes?

 2) The motivation of studying the genomic basis of polymorphism seems rather broad.  Perhaps wing pattern polymorphism?

 3) Adding a reference used for species identification would be useful.

 4) In "Chromosome-scale scaffolds confirmed by the Hi-C data are named in order of size (Figure 2–Figure 5; Table 2)," only Figure 5 and Table 2 seem to be relevant to this information.  Please consider a more suitable place for other figures.

 5) Source of the transcriptomic data used to generate genome annotation was not mentioned.

Are sufficient details of methods and materials provided to allow replication by others?

Yes

Is the rationale for creating the dataset(s) clearly described?

Yes

Are the datasets clearly presented in a useable and accessible format?

Yes

Are the protocols appropriate and is the work technically sound?

Yes

Reviewer Expertise:

evolutionary genomics, phylogenetics, insect evolution

I confirm that I have read this submission and believe that I have an appropriate level of expertise to confirm that it is of an acceptable scientific standard.

# Reviewer report: Reviewer response for version 1

**DOI:** 10.21956/wellcomeopenres.21579.r59464

**Published:** 2023-06-22

**Authors:** Jason Hill

**Affiliations:** 1 Science for Life Laboratory, Department of Medical Biochemistry and Microbiology, Uppsala University, Uppsala, Uppsala County, Sweden

The authors assembled and annotated the genome of the Lichen Button, *Acleris literana. *The methods employed represent an excellent application of the best available methods for generating reference sequences of Lepidoptera. While no particular hypotheses were tested here, this work represents a valuable contribution to the resources available for further work.

 A direct link to the genome and transcriptome sequence would have been appreciated.

Are sufficient details of methods and materials provided to allow replication by others?

Yes

Is the rationale for creating the dataset(s) clearly described?

Yes

Are the datasets clearly presented in a useable and accessible format?

Partly

Are the protocols appropriate and is the work technically sound?

Yes

Reviewer Expertise:

Population genomics

I confirm that I have read this submission and believe that I have an appropriate level of expertise to confirm that it is of an acceptable scientific standard.
